# Special Issue “Molecular Therapeutics for Diabetes and Related Complications”

**DOI:** 10.3390/ijms26125585

**Published:** 2025-06-11

**Authors:** Kota V. Ramana

**Affiliations:** Department of Biomedical Sciences, Noorda College of Osteopathic Medicine, Provo, UT-84606, USA; karamana@noordacom.org

Diabetes mellitus is a significant public health challenge worldwide, and in many developing countries, diabetes and its complications are the number one cause of morbidity and mortality. Besides genetic predisposition, unhealthy lifestyle habits, unhealthy diet, stress, and lack of appropriate physical activity could lead to increased and uncontrolled blood glucose levels, insulin resistance, and obesity, which eventually lead to diabetic mellitus. There are two major types of diabetes. Type 1 diabetes results from genetic predisposition and autoimmune destruction of pancreatic beta cells. Type 2 diabetes is due to increased stress, insulin resistance, obesity, and a sedentary lifestyle. Although hyperglycemia can be controlled with pharmaceutical drugs, lifestyle adjustments, increased physical activity, and reduced stress, the underlying molecular causes of diabetes and its complications still need to be explored. Recent advances in molecular therapeutics target molecular mechanisms involved in glucose metabolism, insulin signaling, and pancreatic β-cell function.

Recent studies suggest that glucagon-like peptide-1 (GLP-1) receptor agonists and dipeptidyl peptidase-4 (DPP-4) inhibitors enhance insulin secretion, offering cardiovascular benefits [1,2]. Similarly, sodium–glucose co-transporter 2 (SGLT 2) inhibitors have been shown to lower blood sugar by promoting urinary glucose excretion and protecting renal and heart health [3]. Gene and stem cell therapies are also emerging, with gene editing tools like clustered regularly interspaced short palindromic repeats (CRISPR) aiming to correct insulin-related defects, and stem cells being used to generate insulin-producing cells [4]. However, conventional treatments such as insulin and glucose-lowering drugs may also come with potential side effects. For example, GLP-1 receptor agonists may cause gastrointestinal issues and, in rare cases, pancreatitis or thyroid concerns [5]. DPP-4 inhibitors could also lead to respiratory symptoms and joint pain [6]. Similarly, SGLT2 inhibitors have been shown to increase the risk of urinary infections and diabetic ketoacidosis [7]. Therefore, careful monitoring and effective dosage adjustment are crucial when using these drugs. Further, recent advances in precision medicine and pharmacogenomics studies could also help to personalize treatment strategies and individual drug responses. Reducing oxidative stress, increasing antioxidant defenses, and modulating the pathways that regulate key molecular pathways in innate immune and inflammatory responses are also emerging approaches to control diabetes and its associated complications such as retinopathy, neuropathy, nephropathy, and cardiovascular diseases. Thus, developing novel therapeutics holds significant promise for controlling diabetes and improving long-term patient outcomes.

In this Special Issue, we compile recent findings on the role of novel molecular therapeutics in ameliorating diabetes and related complications. The compiled review and research articles discuss how current molecular therapeutics represent a paradigm shift in treating diabetes and its complications.

An excellent article by Muntean et al. (contribution 1) investigated whether specific gene polymorphisms associated with type 2 diabetes (ADIPOQ rs266729, CDKN2A/2B rs10811661, and SSR1 rs9505118) are linked to gestational diabetes mellitus (GDM) and perinatal outcomes in Romanian pregnant women. This study reported that among 213 participants, those with GDM had significantly higher pre-pregnancy body mass index (BMI), insulin resistance, and lower adiponectin levels, as well as more frequent gestational hypertension and perineal lacerations. However, no significant associations were found between the studied gene polymorphisms and GDM. Only weak correlations were observed between specific genetic models and certain birth outcomes, suggesting that these polymorphisms are not major contributors to GDM in this population. Similarly, Guardiola et al. (contribution 2) showed the relationship between specific growth differentiation factor 15 (GDF15) gene variants and metabolic diseases, focusing on three SNPs in a cohort of 153 individuals. Among these, only the rs1054564 variant was significantly associated with elevated GDF15 serum levels and a higher frequency of diabetes and atherosclerotic carotid plaque. These studies thus suggest that the rs1054564 variant independently increased the risk of diabetes and subclinical atherosclerosis, suggesting its potential as a genetic marker for these conditions. Further, a 12-year follow-up METSIM cohort study by Fernandes Silva et al. (contribution 3) identified the metabolic markers associated with impaired kidney function by analyzing 1,009 metabolites in 10,159 Finnish men. They found 108 metabolites significantly associated with decreased eGFR, including 28 novel ones across various categories such as amino acids, lipids, and xenobiotics. These findings offer new insights into metabolic pathways linked to kidney function decline and may help in the early detection and prevention of kidney-related complications.

Esteves-Monteiro et al. (contribution 4) examined the gastrointestinal complications of diabetes in type 2 diabetic Goto-Kakizaki (GK) rats. They showed a significant thickening of the intestinal wall and muscular layers and a reduction in myenteric neuronal density, suggesting structural and neurological alterations in GK rats. Diabetic rats showed decreased glutathione (GSH) levels and a lower GSH/GSSG ratio in most gut segments. This indicates that diabetes induces substantial intestinal remodeling and oxidative damage, which may underlie the gastrointestinal dysfunction frequently observed in diabetic patients. Another animal study by Drygalski et al. (contribution 5) showed the potential role of phloroglucinol (PHG) in preventing non-alcoholic fatty liver disease (NAFLD) and insulin resistance caused by a high-fat diet in Wistar rats. PHG treatment improved fasting glucose levels, insulin sensitivity, and liver health. PHG also influenced gastric motility through potassium channel activation and nitric oxide signaling, suggesting PHG as a promising candidate for further clinical investigation.

Interestingly, a case study by Mikami-Saito et al. (contribution 6) reported an association between oral Semaglutide and carnitine depletion. A 34-year-old male with multiple acyl-CoA dehydrogenase deficiency developed hypoglycemia and significantly reduced blood-free carnitine levels after switching from injectable to oral Semaglutide. This finding highlights the need for clinicians to monitor carnitine levels in at-risk patients taking salcaprozate sodium-containing medications.

This Special Issue also published four informative review articles on recent molecular therapeutics in diabetes. Puscasu et al. (contribution 7) discussed the potential of N-methyl-D-aspartate receptor (NMDAR) antagonists such as ketamine, memantine, and methadone as therapeutic options for neuropathic pain. They indicated these agents’ efficacy in managing various forms of neuropathic pain, which is a common diabetes complication. Another article by Cheng and Zhang discussed how mesenchymal stem cell (MSC) therapy is a promising approach for reversing renal damage and restoring kidney function in diabetic kidney disease (DKD). Specifically, they reported current research on MSC-based therapies, their mechanisms of action, and their integration with conventional treatments. Similarly, Guo et al. (contribution 8) discussed recent advancements in molecular therapeutics such as miRNA therapy, stem cell therapy, gene therapy, gut microbiota-targeted treatment, and lifestyle interventions as promising new avenues for DKD management. Finally, a systematic review by Keskesiadou et al. (contribution 9) explored the potential link between endocrine-disrupting chemicals (EDCs) and the development of type 1 diabetes mellitus. This study identified a correlation between type 1 diabetes and specific EDCs, including bisphenol A, bisphenol S, phthalates, dioxins, and persistent organic pollutants (POPs). Thus, this report supports a correlation between EDCs and type 1 diabetes.

In conclusion, the articles published in this Special Issue highlight molecular therapeutics as a transformative approach to treat diabetes and its complications. By targeting the genetic and biochemical pathways involved in the pathophysiology of diabetes, these advanced therapies could significantly improve patient outcomes. Further, continued research is essential to fully understand how these novel treatments will facilitate a future where diabetes is manageable and potentially curable.

## References

[1] Gieroba B., Kryska A., Sroka-Bartnicka A. (2025). Type 2 diabetes mellitus—Conventional therapies and future perspectives in innovative treatment. Biochem. Biophys. Rep..

[2] Bae J.H. (2025). SGLT2 Inhibitors and GLP-1 Receptor Agonists in Diabetic Kidney Disease: Evolving Evidence and Clinical Application. Diabetes Metab. J..

[3] Moustafa B., Trifan G. (2025). The Role of Diabetes and SGLT2 Inhibitors in Cerebrovascular Diseases. Curr. Neurol. Neurosci. Rep..

[4] Arivarasan V.K., Diwakar D., Kamarudheen N., Loganathan K. (2025). Current approaches in CRISPR-Cas systems for diabetes. Prog. Mol. Biol. Transl. Sci..

[5] Xie Y., Choi T., Al-Aly Z. (2025). Mapping the effectiveness and risks of GLP-1 receptor agonists. Nat. Med..

[6] Rai P., Dwibedi N., Rowneki M., Helmer D.A., Sambamoorthi U. (2019). Dipeptidyl Peptidase-4 Inhibitors and Joint Pain: A Retrospective Cohort Study of Older Veterans with Type 2 Diabetes Mellitus. Am. Health Drug Benefits.

[7] Hamblin P.S., Wong R., Ekinci E.I., Fourlanos S., Shah S., Jones A.R., Hare M.J.L., Calder G.L., Epa D.S., George E.M. (2019). SGLT2 Inhibitors Increase the Risk of Diabetic Ketoacidosis Developing in the Community and During Hospital Admission. J. Clin. Endocrinol. Metab..

